# THE ROLE OF TELEHEALTH PAYMENT PARITY ON RECOMMENDED CARE AMONG WORKERS WITH HIGH-RISK FACTORS OF DEMENTIA

**DOI:** 10.1093/geroni/igae098.4372

**Published:** 2024-12-31

**Authors:** Zhang Zhang

**Affiliations:** Johns Hopkins Bloomberg School of Public Health, Baltimore, Maryland, United States

## Abstract

**Objective:**

This study examines the impact of telehealth payment parity, requiring equal payment rate for telehealth and in-person services, on the use of recommended care services and emergency department (E.D.) visits among insured workers with high-risk factors of dementia.

**Methods:**

We conducted a causal inference design and adopted a two-way fixed-effect difference-in-differences approach using the Merative Commercial Claims and Encounters database from 2019 to 2021. We focused on insured workers with mental health disorders or cardiometabolic risks (CMRs), which are high-risk factors for dementia.

**Results:**

Telehealth payment parity significantly increased psychotherapy visits by 0.2208 visits per quarter and tele-psychotherapy by 0.4107 visits per quarter. Additionally, it reduced E.D. visits among those with mental health disorders by 0.003 visits per quarter, a 25% relative decrease. However, the policy did not significantly enhance the uptake of preventive care counseling for CMRs.

**Conclusion:**

Telehealth payment parity effectively boosted psychotherapy usage and reduced E.D. visits for mental health disorders. Public health Implications: Future policies and public health interventions should optimize the telehealth payment model to enhance preventive care uptake, especially for those with CMRs.

